# Prehospital management and outcomes of traumatic cardiac arrest in older adults: a French nationwide registry study

**DOI:** 10.1016/j.resplu.2026.101486

**Published:** 2026-09-10

**Authors:** Axel Benhamed, Baptiste Morcel, Jean-Stephane David, Ewoud Ter Avest, Tobias Gauss, James van Oppen, Karim Tazarourte, Eric Mercier, RéAC investigators, Bages-Limoges Florence, Dhers Marion, Fuster Patrick, Bellow Tobola, Peguet Olivier, Besnier Sylvie, Jardel Benoit, Buleon Clement, Avondo Aurelie, Gillet Stephane, Monchard Franck, Guillaumee Frederic, Jacob Line, Kindle Carine, Marquet Jean-Luc, Watrelot Olivier, Cheminat Claude, Gonzalez Denis, Blottiaux Emmanuel, Branche Fabienne, Delemar Thibault, Ricard-Hibon Agnes, Curunet-Raoul Anne Dominique, Serre Patrice, du Besset Marc, Poincet Geraldine, Lefevre Nathalie, Puchois Aurelien, Leraitre Thierry, Wyckaert Geoffrey, Capel Olivier, Coffin Pierre, Rebet Olivier, Thiriez Sylvain, Pinero David, Revaux Francois, Malbranque Arnaud, Moneron Martine, Dumas Franck, Hellal Meriem, Murgue Davy, Mazille Andre, Jonquet Sebastien, Poupart Patrick, Halbout Laurent, Legendre Kevin, Rouffineau Jean, Ursat Cecile, Kaliszczak Isabelle, Sejourne Gerald, Mesplede Caroline, Leroy Antoine, Dall Acqua David, Genain-Soulier Anne-Sophie, Amizet Loic, Huet Loic, Laborne Francois Xavier, Fievet Marie Laurence, Coletta Mauro, Capitani Georges-Antoine, Forel Alban, Lagadec Steven, Zamparini Guillaume, Beharelle Julien, Orhon Frederic, Harel David, Andregnette Paul, Letarnec Jean Yves, Cicala Jean Francois, Le Pimpec Philippe, Doukhan Mathieu, Raconnat Julien, Martinage Arnaud, Narcisse Sophie, Briole Nicolas, Abarrategui Diego, Line Caroline, Kahn Jean-Philippe, Myriam Van Tricht, Vergne Muriel, Pernot Thomas, Louchet Aurelie, Courcoux Hubert, Prineau Stevens, Bouilleau Guillem, Ta Trung Hung, Bohler Clio, Gonzalez Geraldine, Megy-Michoux Isabelle, Daisey Antoine, Segard Julien, Getti Renaud, Maurel Marion, Bertrand Philippe, Casanova Denis, Peyrat Sylvie, Kuczer Vincent, Agostinucci Jean Marc, Saintonge Stephane, Deuez Florian, Barandiaran Patrick, Lougnon Jean-Paul, Roudiak Nathalie, Meyran Daniel, Boulestein Guillaume, Robert Helene, Laot Melanie, Champenois Vanessa, Chiale Eric, Lacroix Arnaud, Masson Caroline, Rakotonirina Jean-Louis, David Serrano, Champenois Anne, Babin Clement, Wassong Corinne, Sussat Myriam, Bernadet Patricia, Desbuards Claire, Sabbat Sebastien, Gradel Claire, Gosset Pierre, Roland-Billecart Didier, Dupuy Thomas, Bouchel Celia, Joly Marc, Gay Julien, Henry Raymond, Hollecker Eric, Lavaill Loic, Paula Palma Serge, Desaveines Nicolas, Dumouchel Julie, Agbemebia Fabrice, David Olivier, Tabary Romain, Fournier Marc, Garay Emilie, Kadji Roger, Bar Christian, El Abdi Mounir, Deleu Stephanie, Maille Michel, Begaudeau Aurelie, Frances Herve, Alcouffe Fabrice, Randriamalala Solo, Monribot Melanie, Boulanger Bertrand, Payan Anne, Morel Jean-Charles, Dubois Camille, Marchi Jacques, Desclefs Jean-Philippe, Legay Lea, Auger Harold, Spriet Audrey, Anette Bastien, Sadoune Sonia, Durand Olivier, Pes Philippe, Dissait Francois, Jainsky Laure, Vassor Isabelle, Cornaglia Carole, Arnaud Gaelle, Sobocinski Perrine, Legeard Estelle, Paringaux Xavier, Gondret Coralie, Lepage Laurence, Ciocca Philippe, Guenier Pierre-Alban, Bargain Philippe, Gaillard Florence, Vanderstraeten Carine, Simonnet Bruno, Tabarly Julien, Hourdin Nicolas, Duytsche Nicolas, Hamdan David, Grandjon Cyril, Saada Laure, Guillot Philippe, Bredel Stephanie, Emmanuel Vilbois, Goes Frank, Roucaud Nicolas, Lecadet Nathalie, Cwicklinski Emelyne, Menot Pascal, Devillard Arnaud, Minost Jerome, Ruellan Gautier, Cohen Rudy, Grua David, Pavageau Laure, Mahe Aurore, Teyssier Lucie, Fiani Nasri, Vasseur Laurene, Saint Paul Amelie, Malaquin Anne-Sophie, Menay Marion, Rungsawad Phloy, Diez Olivier, Quibel Thomas, Boussarie Catherine, Lamy Pascale, Gaget Matthieu, Majour Gilles, Lancon Virgine, Vidil Elodie, Bouret Marike, Roze Guillaume, Kamga Cyrille, Gelin Emilie, Langeron Frederick, Escutnaire Josephine, Gaillard Nancy, Kottmann Vincent, Rebillard Laurent, Fournier Emmanuelle, Degos Marie-Pierre, Vial Michael, Duconge Antoine, Sanchez Oriana, Thibaud Eric, Reydy Franck, Aubert Gaele, Canon Valentine, Meunier Stephanie, Menu Elsie, Brisson Etienne, Arnaud Aurelie, Clauzel Maxens, Coppin Vincent, Decoster Alice, Egmann Gerald, Cendrie Pascal, Hecker Christine, Remakel Claudine, Guillaume Durand, Dhote Christophe, Desoubeyran Segolene, Delespierre Romain, Lefort Alexandra, Picart Jeanne, Vercher Laurent, Blondet Romain, Marchasson Sebastien, Gautier Lise, Rahmani Raphaelle, Marina Dubois, Foiret Maxime, Leclerc Maxence, Andre Antoine, Bouvier Simon, Treels Justine, Uchlinger Veronique, Ginoux Lucie, Loslier Yannick, Anne Claire Laquerbe, Segard Lionel, Fleurival Kleeve, Corsia Alexandra, Marrakchi Faycal, Labarrere Franck, Roche Florent, Michenet Corinne, Chassery Carine, Dussoulier Sebastien, Dupin Aurelie, Colot Pierre-Etienne, Mascarenc Nathalie, Lomberty Sara, Lemarchand Remy, Bertrand Benoit, Magimel-Pelonnier Edouard, Lespiaucq Christine, Lecoz Julien, Emonet Anne, Katz Elodie, Sigaux Antoine, Carle Olivier, Savineau Jean-Remy, Loubert Richard, Hennache Jonathan, Guegan Annaig, Polini Stephanie, Harle Laure, Lazar Julien, Lefeuvre Stephanie, Messieux Alexis, Caresmel Marion, Beayni Zaher, Dessoy Anne-Laure, Boudard Olivia, Robert Frederique, Fritsch Emmanuelle, Dabri Amira, Moquay Romain, Risser Jean-Dominique, Beaka Placido, Oganov Kirill, Auffret Yannick, Mansouri Nadia, Bazzoli Cyril, Lorain David, Chabannon Margaux, Abdelli Lila, Zitouni Laila, Belin Matthieu, Morel Marechal Emanuel, Caralp Christophe, Bissolokele Pascal, Parsis Pierre, Prouve Christina, Collin Amandine, Jaeger Deborah, De Dinechin Laurene, Daby Ludovic, Hiller Pascale, Dubourg Marie, Pauchet Nicolas, Lambert Julia, Nixdorf Sarah, Florentin Jonathan, Simon Benoit, Chassin Coralie, Vermersch Celine, Jacquemin Renaud, Schmit Anne Catherine, Chadelaud Fabien, Mathieu Benedicte, Gendraud Sarah, Staes Sophie, Campagne Guillaume, Boutin Celia, Olive Stephanie, Faivre-Pierret Caroline, Lefranc Delphine, Dumont Jean-Baptiste, De Schlichting Marie Alix, Mahi Zakaria, Rotival Julie, Quilliet Dominique, Theurey Odile, Duffait Yves, Boursier Marion, Ngonga Marie Yves, Foudi Lahcene, Bonhomme Cecile, Babet Thierry, Lorge Sarah, Poincet Sebastien, Sapir David, Royer Celine, Antonic-Ravaut Celia, Ducasse Pierre, Dubeaux Josephine, Ibrahim Amr, Pangallo Marc Antoine, David Morgane, Ovtcharenko Mariane, Pradel Anne-Lise, Jeziorny Alexandre, Berard Cecile, Desroziers Marie, Pierre Gil, Masse Juliette, Mesli Adil, Galinski Michel, Chapelle Anais, Boyer Romain, Fleury Amandine, Fanchon Armelle, Savu Alexandru, Costa Aurelie, Cahuzac Camille, Klein Amelie, Carruesco Chloe, Chopinaud Pierre-Amaury, Taxis Samuel, Sahuc Benoit, Panharen Pamini, Hammouti Najat, Salignon Julie, Toumert Karim, Barret Morgane, Lacaze Melanie, Conan Pascal, Garden Breche Franck, Guez Charlene, Bontemps Jeremy, Belli Marie-Camille, Roger-Sename Juliette, Coulon Bastien, Mur Sebastien, Hoareau Stephanie, Longo Celine, Billier Laurianne, Jauneau Charline, Hugenschmitt Delphine, Debelle Aurelien, Piccardi Mailis, Lacoste Julien, Deberdt Eric, Le Normand Thomas, Barukh Karen, Allemandet Elodie, Gentilhomme Angelie, Pretalli Jean-Baptiste, Friard Pauline, Sciacca Christelle, Lafay Marina, Bourreau Anna, Chiche Julien, Cattin Vincent, Michelin Florian, Stefanski-Serniclay Virginie, Decker Sandra, Trialoup Sarah Laure, Briatte Apolline, Grigahcine Emma, Lamarche-Vadel Yacine, Viciana Marie, Nenert Eloi, Le Beuan Celine, Evrard Gregoire, Vanparys Ludivine, Djebbar Lynda, Miquelestorena Julie, Lis Laura, Petit Caroline, Beaudoin Adeline, Tabourin Pierre-Emmanuel, Pic Daniel, Dattin Alix, Nave Sophie, Kamara Mariam, Malange Domitille, Rouquet Jimmy, Nussbaum Camille, Evain Yoann, Garcia Lea, Pernin Oceane, Barbery Adele, Lavielle Jeanne, Violeau Mathieu, Pancher Agathe, Petitdemange Olivia, Trouvain Helene, Deslais Benoit, Lucas Anne Sophie, Clauw Emmelyne, Verge Guillaume, Joannesse Guillaume, Vancoillie Gregory, Adam Romain, Duchier Caroline, Sanchez Caroline, Nicolin Leandre, Berthelon Antoine, Cissokho Khadidiatou, Plenier Cecile, Dyani Mohamed, Delbergue Marine, Petitdidier Etienne, Le Pennetier Olivier, Chatenet Guillaume, Chantrel Gauthier, Duperron Yann, Busi Olivia, Scuotto Pierre, Nardy Jean Ely, Barberis Christophe, Dubernat Manon, Villain-Coquet Laurent, Weyer Claire Marie, Oliveira Larissa, Hoff Julie, Ballet Celine, Guery Carole, Merle Heloise, Suhas Pauline, Simisdean Ciprian, Etienne Vincent, Dehouck Marc-Antoine, Bruneau Amandine, Pinel Lauren, Grignon Oceane, Ramaherison Thierry, Dumont Nathalie, Amelon Francois-Xavier, Andre Fanny, Guillon Alain, Majoufre Gwenaelle, Rouet Christelle, Piboule Ludovic, Grave Eric, Rallu Martin, Ahui Terence, Drouard Matthieu, Orange Rodolphe, Blain Stephane, Harguindeguy Alexa, Baina Anne, Boutry Celine, Kamga Clotilde, Constant Samuel, Lartaud Manon, Petitprez Martin, Wurtz-Gress Vassilis, Echeikh Malek, Vincent Rayane, Jeanmasson Yoann, Robart Jean-Christophe, Peixoto Brandon, Guinard Sollweig, Ferquel Martin, Yvetot Quentin, Handwerk Tom, Bollani Maxime, Massacrier Sylvie, Megard Marie Fleur, Javaudin Francois, Meunier Juliette, Larcher Fanny, David Sandy, Leduc Aurelien, Arnaudet Idriss, Ngoyi Natacha, Nicolats Ophelie, Trogoff Bruno, Camboni-Pineau Leila, Gonzalez Lola, Polisset Nathalie, Bujon Cecile, Besserve Paulin, Bouhey Emmanuel, Alba Pierre, Marinoni Heloise, Hsing Priscilia, Roux Nicolas, Pradignac Nicolas, Boulanger Chloe, Chesnoy Marine, Guigon Victor, Lory Thomas, Lafon Jean, Bardelay Romain, Bianchi Roland, Oberlin Mathieu, Parisot Sarah, Beunas Veronique, Charney Alexandre, Edange Claire, Mazzoldi Margherita, Chomono Hendricks, Metzger Jacques, Kirsch Sebastien, Sigarteu Marta, Laboure Anne, Dabbagh Sami, Basty-Ghuysen Marielle, Bois Rozenn, Masse Solene, Viratelle Clelia, Dassonville-Decaudin Coralie, Delattre Thomas

**Affiliations:** a Département d’urgence-Centre de recherche, CHU de Québec-Université Laval, Québec, Québec, Canada; b Service SAMU-Urgences, Hôpital Édouard Herriot, Hospices Civils de Lyon, Lyon, France; c Service d’anesthésie-réanimation, Hôpital Lyon Sud, Hospices Civils de Lyon, Pierre-Bénite, France; d London’s Air Ambulance, London, the United Kingdom of Great Britain and Northern Ireland; e Service d’anesthésie-réanimation, CHU Grenoble Alpes, Grenoble, France; f Centre for Urgent and Emergency Care Research, University of Sheffield, Sheffield S1 4DA, the United Kingdom of Great Britain and Northern Ireland

**Keywords:** Out-of-hospital cardiac arrest, Trauma, Aged, Return of spontaneous circulation, Survival

## Abstract

**Background:**

Traumatic cardiac arrest (TCA) carries an overall poor prognosis, yet the impact of advanced age on prehospital management and outcomes remains poorly characterised. Whether older patients with TCA present distinct clinical profiles or respond differently to resuscitation compared with younger adults is largely unknown.

**Methods:**

We conducted a multicenter cohort study using data from the French National Out-of-Hospital Cardiac Arrest Registry (RéAC). All adult patients (≥18 years) with out-of-hospital TCA were included. Patients were categorised as adults (18–64 years) or older adults (≥65 years). The primary outcome was return of spontaneous circulation (ROSC). Secondary outcomes included survival at hospital admission (D0), 30-day survival (D30), and favourable neurological outcome (Cerebral Performance Category 1–2 at D30). Univariate and multivariable logistic regression analyses were performed. A pre-specified subgroup analysis stratified older patients into three age categories (65–74, 75–84, ≥85 years).

**Results:**

Among 5595 patients (4249 younger adults; 1346 older adults [24.1%]), older patients more often achieved ROSC (22.5% vs 18.6%; OR 1.22, 95%CI 1.03–1.45) but were less likely to survive to 30 days (0.5% vs 1.2%; OR 0.34, 95%CI 0.15–0.81). Survival at hospital admission did not differ after adjustment (16.1% vs 13.3%; OR 1.14, 95%CI 0.95–1.38). Older patients more often sustained a fall (52.2% vs 28.5%) and less often sustained thoracic injuries (43.8% vs 59.6%). They received fewer trauma-specific prehospital interventions, most notably thoracostomy (15.2% vs 23.9%) and fluid resuscitation (28.3% vs 37.4%).

**Conclusions:**

In this large French registry study, older patients with TCA achieved return of spontaneous circulation more often than younger adults but were less likely to survive to 30 days. Age alone should not preclude resuscitative efforts, but a concurrent or precipitating medical cause of arrest should be systematically considered in this population.

## Key messages

### What is already known on this subject

Traumatic cardiac arrest carries a poor prognosis with overall survival rates below 5–8%. Most existing evidence on TCA is derived from predominantly young adult cohorts, and the few studies examining the effect of age have reported conflicting findings.

### What this study adds

In a large French national registry, older TCA patients (≥65 years) had higher ROSC rates than younger adults but significantly lower 30-day survival.

### How this might change practice

Age alone should not discourage resuscitative efforts. Clinicians managing older patients presenting as TCA should systematically consider concurrent or precipitating medical causes of arrest, particularly when injury severity appears low or the mechanism is unclear.

## Introduction

Traumatic cardiac arrest (TCA) occurs when severe injury leads to cardiac standstill through mechanisms including exsanguination, hypoxia, tension pneumothorax, or cardiac tamponade.^1^ Although historically considered a near-uniformly fatal condition, TCA is no longer regarded as futile. Reported prognosis nonetheless remains heterogeneous across studies, depending on case-mix, the cause of arrest, and reporting methods, with survival rates ranging from 2% to 8% in large registry studies.^2^, ^3^, ^4^ Some factors associated with return of spontaneous circulation in TCA have been identified, including non-asystolic initial rhythm and cardiac motion on ultrasound,^5^, ^6^ while the impact of age is still debated. These outcomes have been attributed to advances in prehospital care algorithms that emphasise the rapid identification and correction of reversible causes, including external haemorrhage control, oxygenation, chest decompression, and early haemostatic resuscitation.^7^ However, despite increased adherence to guideline-recommended trauma-specific interventions, their impact on patient-centred outcomes remains uncertain.^3^, ^8^

The global population is ageing rapidly leading to a shift in trauma patient demographics being older with more severe trauma.^9^, ^10^, ^11^ Notably, older individuals are increasingly exposed to mechanisms of injury capable of causing TCA, particularly motor vehicle collisions and falls from height.^9^ However, the epidemiology and outcomes of TCA in older patients remain largely understudied. Analyses based on the Japan Trauma Data Bank reported a counter-intuitive finding: survival to discharge was higher in older adults (≥60 years) compared with non-older patients with TCA even after adjustment for injury severity.^12^ However, the authors only included patients with blunt trauma and did not report long-term outcome. More recently, some authors analysed the US National EMS Information System and similarly found that older adults with TCA had higher rates of ROSC compared with younger adults.^13^ However, they reported no data on prehospital management nor on long-term survival. Yet, these findings raise important questions about the clinical profile of TCA in older patients and whether the current paradigm of TCA management, developed predominantly from young adult cohorts, is equally applicable to older patients. In France, the physician-staffed prehospital emergency medical system (EMS) provides on-scene advanced resuscitation for TCA patients, offering a unique perspective on the prehospital phase of care.^7^

Our primary objective was to compare ROSC rates between younger and older adult patients with out-of-hospital TCA. Secondary objectives were to compare survival at hospital admission (D0), 30-day survival (D30), favourable neurological outcome (CPC 1–2 at D30), prehospital management, and to explore outcomes across older age subgroups.

## Methods

### Study design

We conducted a retrospective multicenter cohort study using prospectively collected data from the RéAC registry. This manuscript was prepared in accordance with the Strengthening the Reporting of Observational Studies in Epidemiology (STROBE) statement.^14^

### Setting and data source

The French prehospital emergency medical system (EMS) operates on a two-tiered model. In cases of out-of-hospital cardiac arrest (OHCA), both a fire brigade ambulance and a mobile medical team are dispatched simultaneously to the scene. A bystander may initiate cardiopulmonary resuscitation (CPR) and, if available, defibrillation using a public-access automated external defibrillator (AED) before EMS arrival, often under the guidance of the emergency dispatcher. The fire brigade typically arrives first, takes over from any bystander, and continues basic life support (BLS). Fire brigade personnel are also trained to perform trauma-specific interventions such as external haemorrhage control, spinal motion restriction, and pelvic stabilisation. The mobile medical team consists of an emergency physician, a nurse, and an ambulance driver, and can be dispatched via either ground or helicopter ambulance. The mobile medical team provides advanced life support (ALS), including advanced airway management, intravenous or intraosseous access, administration of vasopressors, fluid resuscitation, blood transfusion, and, when indicated, surgical procedures such as thoracostomy at the scene.^15^

### Participants

We included all adult patients (≥18 years) registered in the RéAC database with an out-of-hospital traumatic cardiac arrest as defined by Utstein criteria,^16^ including those who died before hospital admission. We excluded cases with spontaneous circulation at mobile medical team arrival, aetiology other than traumatic (medical, asphyxia, overdose, electrocution, drowning) or unknown, burns as primary or contributing cause, signs of irreversible death (rigour mortis, lividity), documented do-not-attempt-resuscitation orders or end-of-life status, and absence of resuscitation by the mobile medical team. We categorised patients into two groups: younger adults (18–64 years) and older adults (≥65 years). The older group was further stratified into three pre-specified subgroups: 65–74, 75–84, and ≥85 years. The threshold of 65 years was chosen because it is the conventional definition of the older adult in trauma epidemiology and in most national trauma and cardiac arrest registries, it corresponds to the age from which geriatric-specific trauma triage criteria are generally applied, and it allows comparison with the previous study of TCA in older adults.^17^

### Variables

Mechanism of trauma was classified as blunt or penetrating. Cause of trauma included motor vehicle accident, fall, firearm injury, and stab wound. The registry did not differentiate falls from standing height from falls from a greater height. Site of injury (head, thoracic, abdominal, limbs) was recorded on the basis of the on-scene clinical assessment by the prehospital physician, without systematic radiological or post-mortem confirmation. Location of arrest was categorised as public (e.g. street or other public space) or private (e.g. home or other indoor domestic setting). Witness status was categorised as unwitnessed, bystander-witnessed, or EMS-witnessed (fire brigade or mobile medical team). Bystander CPR and bystander AED use were documented. Response times for fire brigade and mobile medical team were recorded in minutes. Initial cardiac rhythm was classified as asystole, pulseless electrical activity (PEA), or ventricular fibrillation/ventricular tachycardia (VF/VT). Prehospital interventions recorded included epinephrine administration, external haemorrhage control, fluid resuscitation, blood transfusion, endotracheal intubation, and thoracostomy.

### Outcomes

The primary outcome was return of spontaneous circulation (ROSC). Secondary outcomes were: (1) survival at hospital admission (D0); (2) 30-day survival (D30); and (3) favourable neurological outcome defined as Cerebral Performance Category (CPC) 1–2 at D30.

### Statistical analysis

Categorical variables were expressed as numbers and percentages and continuous variables as medians with interquartile ranges (IQR). Comparisons between groups used chi-squared tests or Fisher’s exact test for categorical variables and Wilcoxon rank-sum tests for continuous variables. Missing covariate data were handled using multiple imputation by chained equations (10 imputed datasets, 5 iterations), with logistic regression for categorical variables and predictive mean matching for continuous variables.

Separate logistic regression models were fitted for each outcome with age group (≥65 vs. <65 years) as the main exposure of interest. Univariate logistic regression was performed to estimate crude odds ratios (ORs) with 95% confidence intervals (CIs). Multivariablee logistic regression was used to estimate adjusted OR (and 95% CI) for potential confounders selected on the basis of prior literature and clinical relevance (sex, mechanism of trauma, site(s) of injury, witness status, bystander CPR and AED use, fire brigade and mobile medical team response times, signs of life and initial cardiac rhythm at mobile medical team arrival as well as prehospital interventions). Complete adjusted estimates for all covariates included in each multivariable model are presented in Supplementary Tables S1–S4. A pre-specified subgroup analysis repeated these analyses within the older age strata (65–74, 75–84, ≥85 years), using the younger adult group (18–64 years) as the reference. As a sensitivity analysis, multivariate logistic regression models were fitted separately on each of the 10 imputed datasets and on the original complete-case dataset. The resulting estimates were compared with the pooled estimates to assess the potential impact of missing data and the imputation procedure on the findings (Supplementary Table S5). A *p*-value < 0.05 was considered statistically significant.

### Ethics

The registry received approval from the French national data protection commission (Commission Nationale de l’Informatique et des Libertés, CNIL) [#910946] and approval from the French advisory committee on information processing in material research in the field of health (Comité Consultatif sur le Traitement de l’Information en Matière de Recherche dans le Domaine de la Santé) [#10.326Ter]. In line with the French legislation on retrospective studies of registry data, patient consent was not required.

## Results

### Population characteristics

A total of 5595 adult patients with out-of-hospital TCA were included (Fig. 1): 4249 (75.9%) in the younger adult group (18–64 years) and 1346 (24.1%) in the older adult group (≥65 years). Within the older adult group, 622 (46.2%) were aged 65–74 years, 489 (36.3%) were aged 75–84 years, and 235 (17.5%) were aged ≥85 years. The median age was 39 years (IQR 27–51) in the younger adult group and 75 years (IQR 70–82) in the older adult group. The proportion of males was lower in older adults (65.4% vs. 80.4%, *p* < 0.001). Blunt trauma was the most common mechanism of injury in both groups (86.7% vs. 78.3%, *p* < 0.001), with falls representing the leading cause in older adults (52.2% vs. 28.5%) and motor vehicle accidents in younger adults (38.5% vs. 50.1%) (*p* < 0.001). Head injuries were slightly more frequent in older adult patients (78.8% vs. 75.8%, *p* = 0.033), while thoracic injuries (43.8% vs. 59.6%, *p* < 0.001), abdominal injuries (26.1% vs. 36.0%, *p* < 0.001), and limb injuries (18.9% vs. 23.5%, *p* < 0.001) were more frequently observed in younger adults. Initial cardiac rhythm did not differ significantly between groups (*p* = 0.258), most patients exhibited non-shockable rhythms: asystole (87.6% vs. 88.7%) or pulseless electrical activity (9.5% vs. 9.9%). However, signs of life were more frequent in older adults (7.1% vs. 5.2%, *p* = 0.010).

**Fig. 1:**
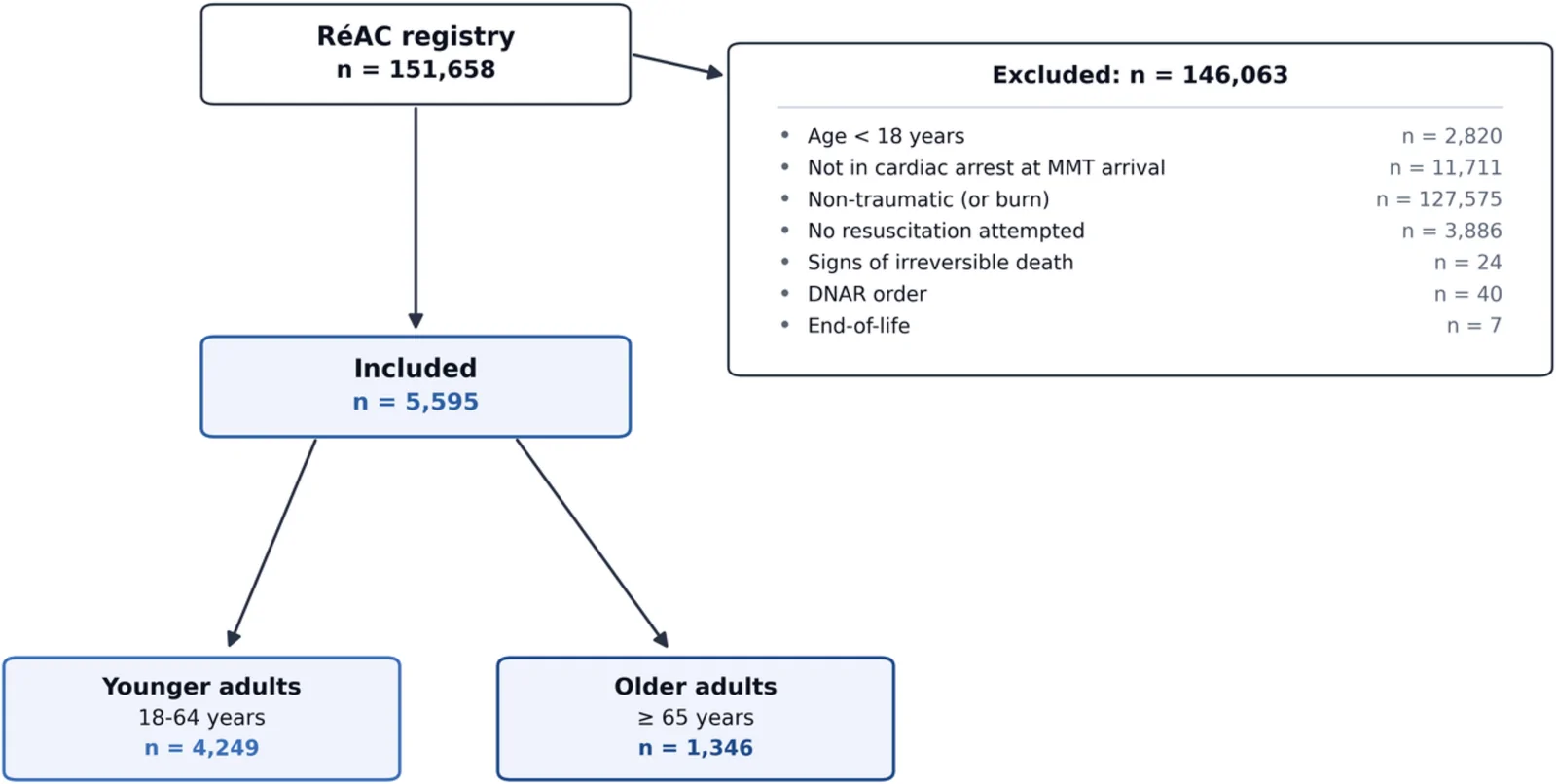
**Flow chart**.

Regarding mobile medical team prehospital management, we found that epinephrine was administered less frequently to older adult patients (94.7% vs. 97.0%, *p* < 0.001), as were fluid resuscitation (28.3% vs. 37.4%, *p* < 0.001), blood transfusion (3.4% vs. 5.0%, *p* = 0.018), intubation (87.5% vs. 91.9%, *p* < 0.001), and thoracostomy (15.2% vs. 23.9%, *p* < 0.001) (Table 1). Subgroups characteristics are presented in Table 2.

**Table 1:** Prehospital characteristics of younger adult and older adult patients with out-of-hospital traumatic cardiac arrest.

	**Younger adults (18–64 years)** ***n* = 4249**	**Older adults (≥65 years)** ***n* = 1346**	***p*-value**
Age, years	39 [27–51]	75 [70–82]	<0.001
Sex			<0.001
Male	3415 (80.4%)	880 (65.4%)	
Female	832 (19.6%)	466 (34.6%)	
Mechanism of trauma			<0.001
Blunt	3327 (78.3%)	1167 (86.7%)	
Penetrating	922 (21.7%)	179 (13.3%)	
Cause of trauma*			<0.001
Motor vehicle accident	1525 (50.1%)	358 (38.5%)	
Fall	866 (28.5%)	485 (52.2%)	
Firearm	328 (10.8%)	69 (7.4%)	
Stab	325 (10.7%)	17 (1.8%)	
***Site of injury***†			
Head	2903 (75.8%)	949 (78.8%)	0.033
Thoracic	2282 (59.6%)	528 (43.8%)	<0.001
Abdominal	1378 (36.0%)	314 (26.1%)	<0.001
Limbs	899 (23.5%)	228 (18.9%)	<0.001
Location			<0.001
Public	3025 (77.6%)	665 (54.6%)	
Private	873 (22.4%)	553 (45.4%)	
Witness status			<0.001
Unwitnessed	914 (21.5%)	385 (28.7%)	
Bystander	2628 (61.9%)	730 (54.4%)	
EMS	702 (16.5%)	228 (17.0%)	
Bystander CPR	1429 (33.6%)	428 (31.8%)	0.515
Bystander AED	14 (0.3%)	5 (0.4%)	0.790
**EMS response times, min**			
Fire brigade	7 [2–10]	7 [3–11]	0.020
Mobile medical team	15 [9–22]	16 [10–24]	0.002
Signs of life	221 (5.2%)	95 (7.1%)	0.010
Initial cardiac rhythm			0.258
Asystole	3767 (88.7%)	1179 (87.6%)	
PEA	404 (9.5%)	133 (9.9%)	
VF/VT	78 (1.8%)	34 (2.5%)	
**Prehospital interventions**			
Epinephrine	4120 (97.0%)	1275 (94.7%)	<0.001
External haemorrhage control	415 (9.8%)	117 (8.7%)	0.242
Fluid resuscitation	1591 (37.4%)	381 (28.3%)	<0.001
Blood transfusion	211 (5.0%)	46 (3.4%)	0.018
Intubation	3903 (91.9%)	1178 (87.5%)	<0.001
Thoracostomy	1015 (23.9%)	205 (15.2%)	<0.001

Categories not mutually exclusive.

AED = automated external defibrillator; CPR = cardiopulmonary resuscitation; EMS = emergency medical services (fire brigade or mobile medical team); IQR = interquartile range; PEA = pulseless electrical activity; VF/VT = ventricular fibrillation/ventricular tachycardia.

Data are *n* (%) or median [IQR].

* Missing: 1205 younger adults, 417 older adults.

† Missing: 418 younger adults, 141 older adults.

**Table 2:** Prehospital characteristics of older adult patients by age subgroup.

	**65–74 years** ***n* = 622**	**75–84 years** ***n* = 489**	**≥85 years** ***n* = 235**	***p*-value**
Age, years	69 [67–72]	79 [77–82]	87 [86–90]	<0.001
Sex				0.081
Male	426 (68.5%)	305 (62.4%)	149 (63.4%)	
Female	196 (31.5%)	184 (37.6%)	86 (36.6%)	
Mechanism of trauma				0.343
Blunt	531 (85.4%)	427 (87.3%)	209 (88.9%)	
Penetrating	91 (14.6%)	62 (12.7%)	26 (11.1%)	
*Cause of trauma*				<0.001
Motor vehicle accident	160 (37.6%)	134 (40%)	64 (37.2%)	
Fall	223 (52.3%)	165 (49.8%)	97 (56%)	
Firearm	37 (8.7%)	22 (6.6%)	10 (5.8%)	
Stab	6 (1.4%)	10 (3.0%)	1 (0.6%)	
***Site of injury***				
Head	426 (77.0%)	346 (79.0%)	177 (83.1%)	0.136
Thoracic	268 (48.5%)	180 (41.1%)	80 (37.6%)	0.010
Abdominal	159 (28.8%)	113 (25.8%)	42 (19.7%)	0.042
Limbs	108 (19.5%)	82 (18.7%)	38 (17.8%)	0.872
Location				0.148
Public	318 (57.2%)	225 (51.0%)	122 (55.2%)	
Private	238 (42.8%)	216 (49.0%)	99 (44.8%)	
Witness status				0.602
Unwitnessed	190 (30.6%)	129 (26.4%)	66 (28.0%)	
Bystander	326 (52.6%)	277 (56.8%)	127 (54.0%)	
EMS	104 (16.8%)	82 (16.8%)	42 (18.0%)	
Bystander CPR	205 (33.0%)	150 (30.7%)	73 (33%)	0.556
Bystander AED	3 (0.6%)	2 (0.5%)	0 (0.0%)	0.851
**EMS response times, min**				
Fire brigade	7 [3–12]	8 [4–12]	6 [3–10]	0.156
Mobile medical team	16 [10–24]	17 [10–25]	16 [9–22]	0.343
Signs of life	37 (5.9%)	37 (7.6%)	21 (8.9%)	0.269
Initial cardiac rhythm				0.445
Asystole	554 (89.1%)	426 (87.1%)	199 (84.7%)	
PEA	53 (8.5%)	52 (10.6%)	28 (12%)	
VF/VT	15 (2.4%)	11 (2.2%)	8 (3.4%)	
**Prehospital interventions**				
Epinephrine	597 (96.0%)	463 (94.7%)	215 (91.5%)	0.052
External haemorrhage control	47 (7.6%)	48 (9.8%)	22 (9.4%)	0.383
Fluid resuscitation	184 (29.6%)	133 (27.2%)	64 (27.2%)	0.629
Blood transfusion	27 (4.3%)	15 (3.1%)	4 (1.7%)	0.143
Intubation	544 (87.5%)	442 (90.4%)	192 (81.7%)	0.002
Thoracostomy	94 (15.1%)	81 (16.6%)	30 (12.8%)	0.409

Data are *n* (%) or median [IQR].

AED = automated external defibrillator; CPR = cardiopulmonary resuscitation; EMS = emergency medical services; IQR = interquartile range; AED = automated external defibrillator; CPR = cardiopulmonary resuscitation; EMS = emergency medical services (fire brigade or mobile medical team); FB = fire brigade; IQR = interquartile range; PEA = pulseless electrical activity; VF/VT = ventricular fibrillation/ventricular tachycardia.; PEA = pulseless electrical activity; VF/VT = ventricular fibrillation/ventricular tachycardia.

### Comparison of patient outcomes

ROSC was achieved in 22.5% (303/1346) of older patients compared with 18.6% (791/4248) of younger patients. Survival at hospital admission (D0) and at 30-days (D30) were respectively 16.1% (217/1345) and 0.5% (7/1346) in the older adult group and 13.3% (565/4249) and 1.2% (51/4249) in the younger adult group. After adjustment, older age was independently associated with a higher rate of ROSC (OR 1.22, 95% CI 1.03–1.45, *p* = 0.019), a similar rate of D0-survival (OR 1.14, 95% CI 0.95–1.38, *p* = 0.161) and a lower rate of D30-survival (OR 0.34, 95% CI 0.15–0.81, *p* = 0.015). Favourable neurological outcome (CPC 1–2 at D30) did not differ significantly between groups, being observed in 5 of 1344 older patients (0.4%) and 23 of 4236 younger adults (0.5%) (OR 0.58, 95% CI 0.19–1.76, *p* = 0.336) (Fig. 2).

**Fig. 2:**
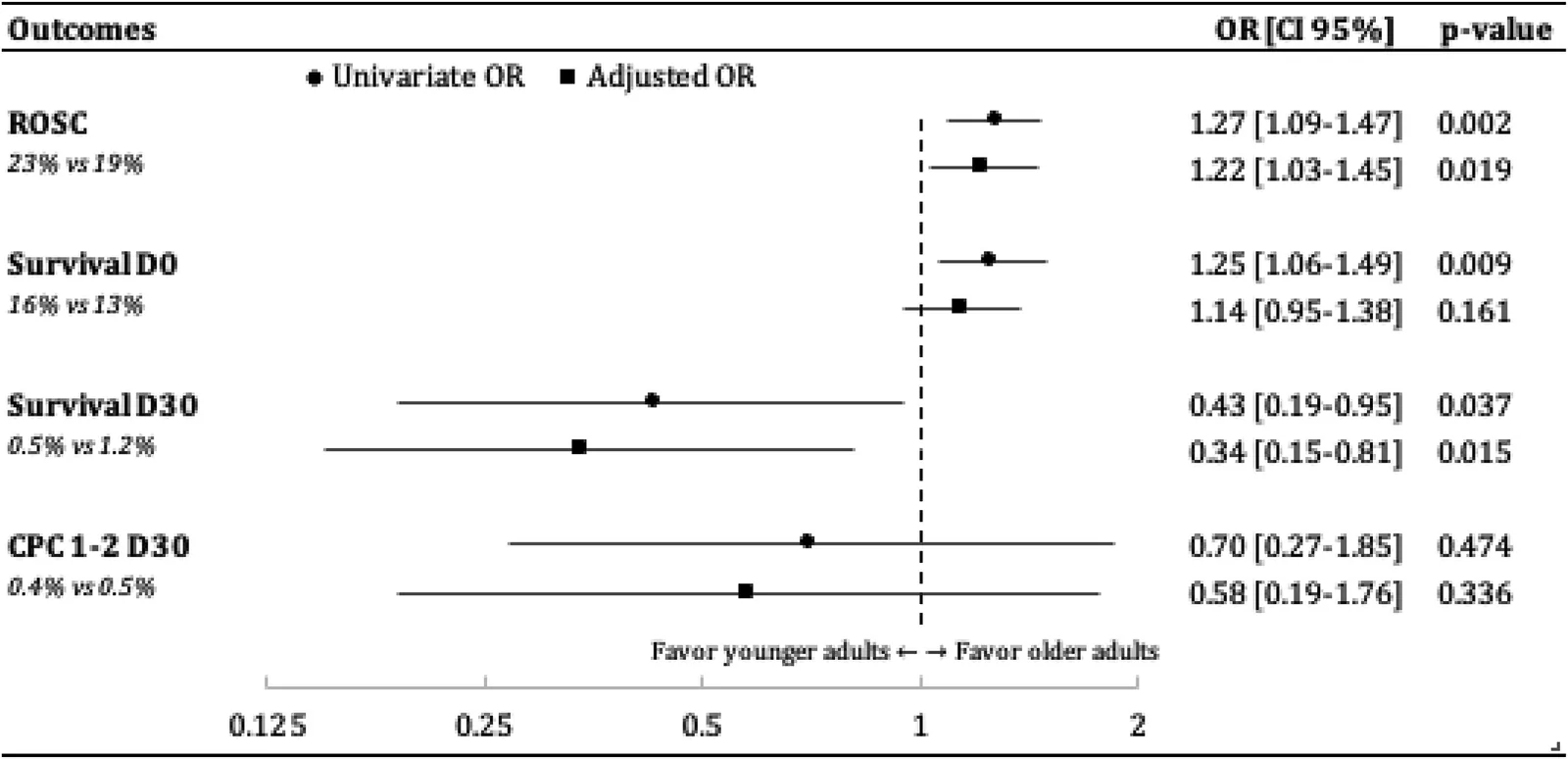
**Forest plot of outcomes in older adults vs younger adults with traumatic cardiac arrest**. Reference: younger adults; Adjustment variables: sex, mechanism of trauma, site(s) of injury, witness status, bystander CPR, bystander AED use, FB response time, MMT response time, signs of life, initial cardiac rhythm, epinephrine, external haemorrhage control, fluid resuscitation, blood transfusion, intubation, thoracostomy. CI = confidence interval; CPC = cerebral performance category; D0 = day 0; D30 = day 30; ROSC = return to spontaneous circulation.

Among older subgroups, the 75–84 year age group showed a higher rate of ROSC (OR 1.41, 95% CI 1.10–1.80, *p* = 0.006) and D0 survival (OR 1.42, 95% CI 1.09–1.86, *p* = 0.010) compared with younger adults. Multivariable models for CPC 1–2 at D30 in the subgroup analysis failed to converge due to small event numbers.

## Discussion

This study, based on a large French national registry, examined the impact of age on prehospital management and outcomes of out-of-hospital TCA. We found that older patients received fewer prehospital invasive interventions and displayed a dissociation between short-term and longer-term outcomes: older age was significantly associated with a higher probability of ROSC but a lower 30-day survival. As detailed below, several clinical and pathophysiological mechanisms, together with differences in care, can plausibly account for this dissociation.

Our findings are consistent with the two existing studies specifically examining age in TCA. Norii et al. reported higher survival to discharge in older adult blunt TCA patients from Japan (5.3% vs. 3.4%; OR 1.58, 95% CI 1.27–1.96), with the older survivor group having notably lower Injury Severity Scores (median 18 vs. 34 in non-survivors).^12^ Our study extends this observation by demonstrating that this apparent advantage at the time of ROSC does not translate into 30-day survival, a finding that could not be assessed in the Japanese study where only survival to discharge was available. Shekhar et al., using the US NEMSIS database, similarly found higher ROSC (11.3% vs. 7.5%) in older adults.^13^ Our study provides the first evidence from a physician-staffed EMS system, where on-scene diagnostic capability is presumably higher than in paramedic-based systems, yet the same dissociation persists.

Several factors may contribute to this apparent paradox. First, the clinical profile of older TCA patients differed substantially from that of younger adults. Older patients more frequently sustained cardiac arrest after lower-energy mechanisms, predominantly falls, and less frequently had thoracic and abdominal injuries, whereas in younger patients out-of-hospital TCA more often followed high-energy or penetrating mechanisms leading to rapidly fatal thoracoabdominal haemorrhage or severe multisystem injury. Importantly, the cause of arrest, rather than the mechanism alone, is a major determinant of the timing of death: exsanguination tends to be rapidly fatal in the prehospital phase, whereas isolated traumatic brain injury more often leads to delayed in-hospital death.^18^ A lower contribution of exsanguination to the cause of arrest in older patients, who less frequently sustain high-energy or penetrating trauma, may therefore favour a higher probability of transient ROSC while shifting mortality to a later, in-hospital phase, a sequence compatible with the lower 30-day survival we observed. In addition, older adults presented more frequently with signs of life and a non-significant trend toward more shockable rhythms, which may preserve some short-term reversibility and further increase the probability of transient ROSC. Finally, manual detection of a pulse or of a low-output state is unreliable in the absence of ultrasound or invasive arterial monitoring^19^; some older patients categorised as being in cardiac arrest may in fact have had a profound low-flow state rather than true mechanical arrest, which could facilitate the recovery or maintenance of a spontaneous circulation while leaving cerebral perfusion critically impaired and thereby contribute to poor longer-term outcomes. However, ROSC in this context does not necessarily translate into meaningful survival, as older patients are more vulnerable to post-resuscitation shock, hypoxic-ischemic brain injury, coagulopathy, reduced physiological reserve, and treatment limitation decisions.^20^ Thus, age may operate less as a determinant of immediate resuscitation success than as a determinant of post-ROSC trajectory and longer-term survival. This interpretation is consistent with the observed dissociation between ROSC and 30-day survival and suggests that, in older TCA patients, ROSC should not be interpreted as a sufficient marker of prognosis without considering injury pattern, pre-arrest physiology, frailty, and early post-resuscitation evolution.

However, the hypothesis raised by Norii et al. in their analysis of the Japanese Trauma Data Bank, suggesting that the observed higher survival in older adult TCA may reflect the inclusion of medical cardiac arrests misclassified as traumatic cannot be excluded.^12^ This misclassification may operate in both directions: a medical event, such as syncope, an arrhythmia, hypoglycaemia, or sepsis, may precipitate a fall and a subsequent arrest that is then labelled traumatic, while conversely the extent of injuries is frequently under-recognised in older patients, in whom major trauma is notoriously underestimated. As falls are often multifactorial, the initial response to resuscitation may in part reflect treatment of the precipitating medical condition rather than of the injury itself. We cannot exclude a similar misclassification in our database, and the absence of comorbidity data prevents its direct assessment. Differences in prehospital management were also observed: older patients received fewer invasive interventions, including less fluid resuscitation, fewer blood transfusions, lower intubation rates, and substantially fewer thoracostomies. Epinephrine, by contrast, was administered to the large majority of patients in both age groups, in keeping with its routine use during advanced life support irrespective of the cause of arrest, so that the age-related differences concerned trauma-specific procedures rather than standard resuscitation drugs. These differences may reflect a genuine reduction in the intensity of care, including explicit or implicit limitation of resuscitative effort in patients perceived as frail or unlikely to survive, which could itself contribute to the lower longer-term survival observed. Consistent with this, Spering et al. reported in a large trauma cohort that severely injured older patients are managed with a more “wait and see” approach, resulting in higher mortality rates.^21^ Alternatively, and not mutually exclusively, the lower-energy injury pattern and the lower prevalence of penetrating or thoracoabdominal trauma in older patients may require fewer time-critical invasive procedures, so that part of the observed difference may reflect appropriate tailoring of care rather than under-treatment.

From a clinical perspective, this study provides evidence that older age alone should not discourage the initiation of resuscitative efforts in TCA. Although 30-day survival was very low in older patients, it was not futile, and favourable neurological outcomes were observed among survivors as reported elsewhere.^21^ However, a favourable 30-day outcome was achieved in only 7 of more than 1300 older patients, so such outcomes remain exceptional; the potential for non-beneficial or overly intensive treatment must therefore be weighed against the small probability of meaningful recovery, and a frailty-attuned approach together with time-limited trials of treatment may help to balance these considerations. Second, clinicians should maintain a high index of suspicion for a precipitating or concurrent medical cause of cardiac arrest when managing older trauma patients, particularly when the mechanism is a fall, apparent injuries are minor, or the initial rhythm is shockable, and should adopt a comprehensive, frailty-attuned assessment rather than settling on a single diagnosis. In such situations, integrating standard ACLS measures (including defibrillation when indicated) alongside the trauma-specific resuscitation algorithm may be appropriate. Third, the finding that fewer invasive prehospital interventions were performed in older patients warrants further investigation to determine whether it represents appropriate tailoring of care or potentially modifiable under-treatment.

Several avenues for future investigation emerge from these findings. First, a prospective study incorporating systematic recording of initial cardiac rhythm, point-of-care echocardiography, and post-mortem computed tomography would help quantify the proportion of medical cardiac arrests misclassified as TCA in older patients. Second, linkage of prehospital TCA registry data with hospital databases (including anatomic trauma severity scoring systems, comorbidities, and in-hospital management) would enable more robust adjustment for potential confounders and assessment of the in-hospital phase of care. Third, a multicenter study comparing TCA outcomes in older patients across physician-staffed and paramedic-based EMS systems would clarify whether the prehospital care model modifies the age-outcome relationship. Fourth, the development and validation of a clinical decision rule to guide the choice between trauma-specific and medical cardiac arrest algorithms in older TCA patients could have direct clinical utility. Fifth, qualitative research exploring clinician decision-making regarding the intensity of resuscitative interventions in older TCA patients could identify potential sources of age-based treatment variation.

### Limitations

Several limitations must be acknowledged. First, as a registry-based study, the analysis is constrained by the variables collected. Notably, we lacked data on injury severity scoring (e.g. ISS, AIS), comorbidities, anticoagulant or antiplatelet use, and prehospital ultrasound findings. These unmeasured confounders may influence the observed associations. Second, patients in whom no resuscitation was attempted by the mobile medical team were excluded; if resuscitation was less frequently attempted in apparently frail older patients judged unlikely to survive, this exclusion could introduce a selection bias whose direction is difficult to anticipate, and a formal analysis of the missingness and of the characteristics of these excluded cases would help to quantify its potential impact.^22^ Third, we did not have data on in-hospital management, which may independently affect outcomes. Notably, decisions regarding trauma team activation, damage-control interventions, intensive care admission, neurosurgical procedures, transfusion strategies, and limitation or withdrawal of life-sustaining therapies may differ according to age, frailty, perceived prognosis, comorbidities, and patient preferences.^23^, ^24^, ^25^, ^26^ Because these variables were not captured in the registry, we could not determine whether the poorer longer-term outcomes observed among older patients reflected irreversible injury severity, reduced physiological reserve, differential in-hospital treatment intensity, or earlier transition to comfort-oriented care. Finally, the adjusted analysis at 30-day should be interpreted with caution because of the limited number of survival events, particularly among older patients (7 survivors and 5 with CPC 1–2) which may have affected model stability and increased the risk of overfitting. However, covariates were selected a priori based on clinical relevance and previous literature to limit data-driven model selection.

## Conclusion

In this large French national registry study, older patients with out-of-hospital traumatic cardiac arrest achieved return of spontaneous circulation more often than younger adults but were significantly less likely to survive to 30 days. This dissociation is compatible with several mechanisms, including a different injury profile, greater vulnerability to post-resuscitation injury, differences in the intensity of prehospital and in-hospital care, and the possible inclusion of medical cardiac arrests misclassified as traumatic. Age alone should not preclude resuscitative efforts, but clinicians should systematically consider a concurrent or precipitating medical cause of arrest, particularly in older patients with low-energy mechanisms, minor apparent injuries, or a shockable rhythm.

## Declaration of generative AI and AI-assisted technologies in the manuscript preparation process

During the preparation of this work the authors used Claude (Anthropic) in order to assist with language editing. After using this tool, the authors reviewed and edited the content as needed and take full responsibility for the content of the published article.

## CRediT authorship contribution statement

**Axel Benhamed:** Writing – original draft, Validation, Methodology, Investigation, Formal analysis, Conceptualization, Supervision. **Baptiste Morcel:** Writing – review & editing, Formal analysis, Data curation, Conceptualization. **Jean-Stephane David:** Writing – review & editing, Methodology, Resources. **Ewoud Ter Avest:** Writing – review & editing, Methodology. **Tobias Gauss:** Writing – review & editing, Supervision. **James van Oppen:** Writing – review & editing, Methodology. **Karim Tazarourte:** Writing – review & editing, Investigation, Resources. **Eric Mercier:** Writing – review & editing, Validation.

## Funding

The RéAC registry is supported by the Société Française de Médecine d’Urgence (SFMU), the Fédération Française de Cardiologie (FFC), the Mutuelle Générale de l’Éducation Nationale (MGEN), the University of Lille, and the Institute of Health Engineering of Lille. The authors declare that the sources of funding had no role in the conduct, analysis, interpretation, or writing of this manuscript.

## Data availability

Data were obtained from the RéAC registry and are available upon reasonable request and approval from the RéAC steering committee.

## Declaration of competing interest

The authors declare that they have no known competing financial interests or personal relationships that could have appeared to influence the work reported in this paper.

## Appendix A Supplementary material

Supplementary Tables Table S1: multivariable logistic regression analyis for returm of spontaneous circulation (ROSC). Table S2: multivariable logistic regression analysis for survival at day 0. Table S3: multivariable logistic regression for 30-day survival. Table S4: multivariable logistic regression for CPC outcome at day 30. Table S5: sensitivity analyses of adjusted associations using complete-case and multiple-imputation datasets.
