# Supplementary material for: Prehospital management and outcomes of traumatic cardiac arrest in older adults: a French nationwide registry study
Source: Resusc Plus. 2026 Sep 10;32:101486. doi: 10.1016/j.resplu.2026.101486 (PMC13629317; doi:10.1016/j.resplu.2026.101486)
Supplement: Supplementary file 1 [file mmc1.docx]

Supplementary Table S1. Multivariable logistic regression analysis for return of spontaneous circulation (ROSC)

| **Characteristic** | **Adjusted OR** | **95% CI** | **p-value** |
| --- | --- | --- | --- |
| **Older age group** | | | |
| No | — | — |  |
| Yes | 1.22 | 1.03, 1.45 | 0.019 |
| **Sex** | | | |
| Male | — | — |  |
| Female | 0.93 | 0.78, 1.10 | 0.386 |
| **Mechanism** | | | |
| Blunt | — | — |  |
| Penetrating | 0.58 | 0.47, 0.71 | **<0.001** |
| **Head injury** | | | |
| No | — | — |  |
| Yes | 1.00 | 0.82, 1.23 | 0.983 |
| **Thoracic injury** | | | |
| No | — | — |  |
| Yes | 0.66 | 0.56, 0.79 | **<0.001** |
| **Abdominal injury** | | | |
| No | — | — |  |
| Yes | 0.72 | 0.60, 0.86 | **<0.001** |
| **Limb injury** | | | |
| No | — | — |  |
| Yes | 0.55 | 0.45, 0.68 | **<0.001** |
| **Witnessed status** | | | |
| Unwitnessed | — | — |  |
| Bystander-witnessed | 1.42 | 1.16, 1.72 | **<0.001** |
| EMS-witnessed | 1.73 | 1.27, 2.37 | **<0.001** |
| **Bystander CPR** | | | |
| No | — | — |  |
| Yes | 1.04 | 0.88, 1.23 | 0.665 |
| **Bystander AED** | | | |
| No | — | — |  |
| Yes | 1.41 | 0.42, 4.73 | 0.568 |
| SP delay | 0.99 | 0.97, 1.01 | 0.410 |
| SMUR delay | 0.99 | 0.98, 1.00 | 0.189 |
| **Signs of life** | | | |
| No | — | — |  |
| Yes | 1.40 | 1.06, 1.86 | 0.020 |
| **Initial rhythm** | | | |
| Asystole | — | — |  |
| PEA | 1.74 | 1.39, 2.17 | **<0.001** |
| VF/VT | 2.49 | 1.64, 3.79 | **<0.001** |
| **Adrenaline** | | | |
| No | — | — |  |
| Yes | 1.29 | 0.83, 1.99 | 0.263 |
| **Orotracheal intubation** | | | |
| No | — | — |  |
| Yes | 0.55 | 0.44, 0.70 | **<0.001** |
| **Expansion / fluid resuscitation** | | | |
| No | — | — |  |
| Yes | 3.56 | 3.05, 4.16 | **<0.001** |
| **Transfusion** | | | |
| No | — | — |  |
| Yes | 2.55 | 1.91, 3.39 | **<0.001** |
| **Hemostatic intervention** | | | |
| No | — | — |  |
| Yes | 1.56 | 1.24, 1.95 | **<0.001** |
| **Thoracotomy** | | | |
| No | — | — |  |
| Yes | 0.47 | 0.38, 0.57 | **<0.001** |

**Notes.** OR, odds ratio; CI, confidence interval; CPR, cardiopulmonary resuscitation; AED, automated external defibrillator; EMS, emergency medical services; PEA, pulseless electrical activity; VF/VT, ventricular fibrillation/ventricular tachycardia. An em dash indicates the reference category. Continuous predictors are reported per unit increase as specified in the main manuscript.

Supplementary Table S2. Multivariable logistic regression analysis for survival at Day 0

| **Characteristic** | **Adjusted OR** | **95% CI** | **p-value** |
| --- | --- | --- | --- |
| **Older age group** | | | |
| No | — | — |  |
| Yes | 1.14 | 0.95, 1.38 | 0.161 |
| **Sex** | | | |
| Male | — | — |  |
| Female | 0.94 | 0.77, 1.14 | 0.525 |
| **Mechanism** | | | |
| Blunt | — | — |  |
| Penetrating | 0.51 | 0.40, 0.64 | **<0.001** |
| **Head injury** | | | |
| No | — | — |  |
| Yes | 1.01 | 0.80, 1.28 | 0.942 |
| **Thoracic injury** | | | |
| No | — | — |  |
| Yes | 0.60 | 0.49, 0.73 | **<0.001** |
| **Abdominal injury** | | | |
| No | — | — |  |
| Yes | 0.68 | 0.55, 0.85 | **<0.001** |
| **Limb injury** | | | |
| No | — | — |  |
| Yes | 0.47 | 0.37, 0.60 | **<0.001** |
| **Witnessed status** | | | |
| Unwitnessed | — | — |  |
| Bystander-witnessed | 1.31 | 1.05, 1.63 | 0.016 |
| EMS-witnessed | 1.54 | 1.09, 2.16 | 0.013 |
| **Bystander CPR** | | | |
| No | — | — |  |
| Yes | 1.09 | 0.90, 1.33 | 0.358 |
| **Bystander AED** | | | |
| No | — | — |  |
| Yes | 1.55 | 0.46, 5.20 | 0.470 |
| SP delay | 0.99 | 0.97, 1.02 | 0.492 |
| SMUR delay | 0.99 | 0.98, 1.00 | 0.224 |
| **Signs of life** | | | |
| No | — | — |  |
| Yes | 1.28 | 0.93, 1.76 | 0.123 |
| **Initial rhythm** | | | |
| Asystole | — | — |  |
| PEA | 1.58 | 1.23, 2.03 | **<0.001** |
| VF/VT | 2.16 | 1.38, 3.38 | **<0.001** |
| **Adrenaline** | | | |
| No | — | — |  |
| Yes | 0.86 | 0.55, 1.35 | 0.511 |
| **Orotracheal intubation** | | | |
| No | — | — |  |
| Yes | 0.69 | 0.52, 0.90 | 0.007 |
| **Expansion / fluid resuscitation** | | | |
| No | — | — |  |
| Yes | 3.56 | 2.99, 4.25 | **<0.001** |
| **Transfusion** | | | |
| No | — | — |  |
| Yes | 2.79 | 2.06, 3.76 | **<0.001** |
| **Hemostatic intervention** | | | |
| No | — | — |  |
| Yes | 1.33 | 1.03, 1.72 | 0.030 |
| **Thoracotomy** | | | |
| No | — | — |  |
| Yes | 0.40 | 0.31, 0.51 | **<0.001** |

**Notes.** OR, odds ratio; CI, confidence interval; CPR, cardiopulmonary resuscitation; AED, automated external defibrillator; EMS, emergency medical services; PEA, pulseless electrical activity; VF/VT, ventricular fibrillation/ventricular tachycardia. An em dash indicates the reference category. Continuous predictors are reported per unit increase as specified in the main manuscript.

Supplementary Table S3. Multivariable logistic regression analysis for 30-day survival

| **Characteristic** | **Adjusted OR** | **95% CI** | **p-value** |
| --- | --- | --- | --- |
| **Older age group** | | | |
| No | — | — |  |
| Yes | 0.34 | 0.15, 0.81 | 0.015 |
| **Sex** | | | |
| Male | — | — |  |
| Female | 1.06 | 0.55, 2.05 | 0.859 |
| **Mechanism** | | | |
| Blunt | — | — |  |
| Penetrating | 0.72 | 0.35, 1.48 | 0.376 |
| **Head injury** | | | |
| No | — | — |  |
| Yes | 0.47 | 0.24, 0.89 | 0.022 |
| **Thoracic injury** | | | |
| No | — | — |  |
| Yes | 0.89 | 0.48, 1.67 | 0.726 |
| **Abdominal injury** | | | |
| No | — | — |  |
| Yes | 0.58 | 0.30, 1.14 | 0.113 |
| **Limb injury** | | | |
| No | — | — |  |
| Yes | 1.10 | 0.56, 2.16 | 0.780 |
| **Witnessed status** | | | |
| Unwitnessed | — | — |  |
| Bystander-witnessed | 3.00 | 0.89, 10.2 | 0.077 |
| EMS-witnessed | 5.45 | 1.27, 23.4 | 0.023 |
| **Bystander CPR** | | | |
| No | — | — |  |
| Yes | 1.29 | 0.61, 2.72 | 0.508 |
| **Bystander AED** | | | |
| No | — | — |  |
| Yes | 4.78 | 0.43, 53.5 | 0.204 |
| SP delay | 0.99 | 0.90, 1.10 | 0.894 |
| SMUR delay | 1.0 | 0.96, 1.03 | 0.781 |
| **Signs of life** | | | |
| No | — | — |  |
| Yes | 3.67 | 1.85, 7.29 | **<0.001** |
| **Initial rhythm** | | | |
| Asystole | — | — |  |
| PEA | 1.98 | 0.98, 4.01 | 0.058 |
| VF/VT | 9.34 | 3.90, 22.4 | **<0.001** |
| **Adrenaline** | | | |
| No | — | — |  |
| Yes | 0.12 | 0.05, 0.28 | **<0.001** |
| **Orotracheal intubation** | | | |
| No | — | — |  |
| Yes | 0.55 | 0.24, 1.25 | 0.155 |
| **Expansion / fluid resuscitation** | | | |
| No | — | — |  |
| Yes | 3.01 | 1.61, 5.62 | **<0.001** |
| **Transfusion** | | | |
| No | — | — |  |
| Yes | 1.73 | 0.76, 3.95 | 0.192 |
| **Hemostatic intervention** | | | |
| No | — | — |  |
| Yes | 1.89 | 0.92, 3.89 | 0.085 |
| **Thoracotomy** | | | |
| No | — | — |  |
| Yes | 0.16 | 0.05, 0.54 | 0.003 |

**Notes.** OR, odds ratio; CI, confidence interval; CPR, cardiopulmonary resuscitation; AED, automated external defibrillator; EMS, emergency medical services; PEA, pulseless electrical activity; VF/VT, ventricular fibrillation/ventricular tachycardia. An em dash indicates the reference category. Continuous predictors are reported per unit increase as specified in the main manuscript.

Supplementary Table S4. Multivariable logistic regression analysis for CPC outcome at Day 30

| **Characteristic** | **Adjusted OR** | **95% CI** | **p-value** |
| --- | --- | --- | --- |
| **Older age group** | | | |
| No | — | — |  |
| Yes | 0.58 | 0.19, 1.76 | 0.336 |
| **Sex** | | | |
| Male | — | — |  |
| Female | 1.19 | 0.44, 3.20 | 0.734 |
| **Mechanism** | | | |
| Blunt | — | — |  |
| Penetrating | 0.42 | 0.13, 1.36 | 0.149 |
| **Head injury** | | | |
| No | — | — |  |
| Yes | 0.29 | 0.10, 0.80 | 0.017 |
| **Thoracic injury** | | | |
| No | — | — |  |
| Yes | 0.77 | 0.27, 2.16 | 0.615 |
| **Abdominal injury** | | | |
| No | — | — |  |
| Yes | 0.21 | 0.05, 0.84 | 0.027 |
| **Limb injury** | | | |
| No | — | — |  |
| Yes | 1.16 | 0.38, 3.50 | 0.796 |
| **Witnessed status** | | | |
| Unwitnessed | — | — |  |
| Bystander-witnessed | 3.07 | 0.38, 25.1 | 0.296 |
| EMS-witnessed | 2.15 | 0.18, 25.2 | 0.542 |
| **Bystander CPR** | | | |
| No | — | — |  |
| Yes | 1.55 | 0.48, 5.02 | 0.461 |
| **Bystander AED** | | | |
| No | — | — |  |
| Yes | 10.5 | 0.46, 238 | 0.138 |
| SP delay | 0.96 | 0.81, 1.14 | 0.661 |
| SMUR delay | 0.93 | 0.85, 1.01 | 0.097 |
| **Signs of life** | | | |
| No | — | — |  |
| Yes | 3.81 | 1.45, 9.99 | 0.007 |
| **Initial rhythm** | | | |
| Asystole | — | — |  |
| PEA | 4.63 | 1.67, 12.8 | 0.003 |
| VF/VT | 23.1 | 7.40, 71.8 | **<0.001** |
| **Adrenaline** | | | |
| No | — | — |  |
| Yes | 0.12 | 0.03, 0.47 | 0.002 |
| **Orotracheal intubation** | | | |
| No | — | — |  |
| Yes | 1.04 | 0.21, 5.07 | 0.964 |
| **Expansion / fluid resuscitation** | | | |
| No | — | — |  |
| Yes | 1.62 | 0.62, 4.22 | 0.319 |
| **Transfusion** | | | |
| No | — | — |  |
| Yes | 3.19 | 1.04, 9.76 | 0.043 |
| **Hemostatic intervention** | | | |
| No | — | — |  |
| Yes | 2.59 | 0.89, 7.60 | 0.082 |
| **Thoracotomy** | | | |
| No | — | — |  |
| Yes | 0.24 | 0.05, 1.21 | 0.084 |

**Notes.** OR, odds ratio; CI, confidence interval; CPR, cardiopulmonary resuscitation; AED, automated external defibrillator; EMS, emergency medical services; PEA, pulseless electrical activity; VF/VT, ventricular fibrillation/ventricular tachycardia. An em dash indicates the reference category. Continuous predictors are reported per unit increase as specified in the main manuscript.

**Supplementary Table S5. Multivariable logistic regression analysis for 30-day survivalSensitivity analyses of adjusted associations using complete-case and multiple-imputation datasets**

|  | **ROSC** | **Survival D0** | **Survival D30** | **CPC 1-2 D30** |
| --- | --- | --- | --- | --- |
| **Number of  complete cases** | 2343 | 2342 | 2342 | 2332 |
| **Complete cases analysis** | 1.26 [0.97-1.63],  p = 0.078 | 1.01 [0.75-1.36],  p = 0.948 | NA | NA |
| **Imputed dataset 1** | 1.23 [1.04-1.46],  p = 0.015 | 1.14 [0.95-1.38],  p = 0.164 | 0.32 [0.12-0.73],  p = 0.011 | 0.52 [0.15-1.53],  p = 0.270 |
| **Imputed dataset 2** | 1.22 [1.03-1.44],  p = 0.020 | 1.14 [0.95-1.38],  p = 0.165 | 0.36 [0.14-0.79],  p = 0.018 | 0.58 [0.17-1.63],  p = 0.331 |
| **Imputed dataset 3** | 1.22 [1.03-1.45],  p = 0.018 | 1.15 [0.95-1.38],  p = 0.152 | 0.35 [0.14-0.76],  p = 0.015 | 0.65 [0.21-1.76],  p = 0.418 |
| **Imputed dataset 4** | 1.23 [1.04-1.45],  p = 0.017 | 1.16 [0.96-1.40],  p = 0.133 | 0.35 [0.14-0.79],  p = 0.018 | 0.60 [0.18-1.69],  p = 0.362 |
| **Imputed dataset 5** | 1.22 [1.03-1.44],  p = 0.020 | 1.14 [0.95-1.38],  p = 0.163 | 0.35 [0.14-0.78],  p = 0.017 | 0.64 [0.20-1.77],  p = 0.420 |
| **Imputed dataset 6** | 1.23 [1.04-1.46],  p = 0.015 | 1.16 [0.96-1.40],  p = 0.134 | 0.35 [0.14-0.77],  p = 0.015 | 0.57 [0.17-1.58],  p = 0.308 |
| **Imputed dataset 7** | 1.21 [1.03-1.44],  p = 0.024 | 1.14 [0.94-1.37],  p = 0.185 | 0.35 [0.13-0.77],  p = 0.017 | 0.58 [0.17-1.66],  p = 0.345 |
| **Imputed dataset 8** | 1.23 [1.04-1.45],  p = 0.016 | 1.15 [0.95-1.38],  p = 0.157 | 0.35 [0.13-0.77],  p = 0.015 | 0.58 [0.17-1.63],  p = 0.330 |
| **Imputed dataset 9** | 1.23 [1.03-1.45],  p = 0.018 | 1.14 [0.94-1.37],  p = 0.176 | 0.36 [0.14-0.80],  p = 0.019 | 0.65 [0.19-1.82],  p = 0.436 |
| **Imputed dataset 10** | 1.23 [1.04-1.45],  p = 0.017 | 1.14 [0.95-1.38],  p = 0.161 | 0.34 [0.13-0.75],  p = 0.013 | 0.51 [0.15-1.47],  p = 0.243 |
| **Pooled analysis** | 1.22 [1.03-1.45],  p = 0.019 | 1.14 [0.95-1.38],  p = 0.161 | 0.34 [0.15-0.81],  p = 0.015 | 0.58 [0.19-1.76],  p = 0.336 |

Sensitivity analyses supported the overall robustness of the findings to missing covariate data, with estimates remaining highly consistent across the 10 imputed datasets for all outcomes. For ROSC and D0-survival, effect estimates were broadly comparable between the complete-case and multiple imputation analyses, although the substantial reduction in sample size in the complete-case analysis led to wider confidence intervals and differences in statistical significance. For D30-outcomes, the complete-case models failed to converge, likely owing to the substantial loss of observations and the limited number of events relative to the number of model parameters.
